# CCT020312 Inhibits Triple-Negative Breast Cancer Through PERK Pathway-Mediated G1 Phase Cell Cycle Arrest and Apoptosis

**DOI:** 10.3389/fphar.2020.00737

**Published:** 2020-05-19

**Authors:** Xiaoli Li, Xiaoping Yu, Duanfang Zhou, Bo Chen, Wenjun Li, Xiangru Zheng, Hongfang Zeng, Liangyuan Long, Weiying Zhou

**Affiliations:** ^1^Department of Pharmacology, College of Pharmacy, Chongqing Medical University, Chongqing, China; ^2^Chongqing Key Laboratory of Drug Metabolism, Chongqing Medical University, Chongqing, China; ^3^Key Laboratory for Biochemistry and Molecular Pharmacology of Chongqing, Chongqing Medical University, Chongqing, China; ^4^Department of Neurology, The Third Affiliated Hospital of Chongqing Medical University (Gener Hospital), Chongqing, China; ^5^Department of Pharmacy, The Third Affiliated Hospital of Chongqing Medical University (Gener Hospital), Chongqing, China; ^6^Department of Gastrointestinal Surgery, The First Affiliated Hospital of Chongqing Medical University, Chongqing, China

**Keywords:** triple-negative breast cancer, CCT020312, protein kinase RNA-like endoplasmic reticulum kinase, apoptosis, cell cycle arrest

## Abstract

Triple-negative breast cancer (TNBC) has a poor prognosis due to the lack of specific therapeutic targets. CCT020312, a selective eukaryotic translation initiation factor 2 alpha (eIF2α)/protein kinase RNA-like endoplasmic reticulum kinase (PERK) activator, may have a potent anti-tumor effect. In the present study, we examined the effects of CCT020312 on TNBC and explored the underlying mechanism. We found that CCT020312 inhibited the viability of TNBC cell lines, MDA-MB-453 and CAL-148, by inducing apoptosis and G1 phase cell cycle arrest. CCT020312 decreased the protein levels of cyclin-dependent kinase 4 (CDK4), CDK6, cyclin D1, and B-cell lymphoma 2 (Bcl-2) and increased the levels of Bcl-2-associated X protein (Bax) and cleaved poly (ADP-ribose) polymerase (PARP) compared with those in the control. CCT020312 activated PERK/eIF2α/activating transcription factor 4 (ATF4)/CCAAT-enhancer binding protein (C/EBP) homologous protein transcription factor (CHOP) signaling and inhibited protein kinase B (AKT)/mammalian target of rapamycin (mTOR) signaling. Furthermore, CCT020312 inhibited tumor growth in an MDA-MB-453 orthotopic xenograft mouse model by activating the PERK/eIF2α/ATF4/CHOP pathway and inhibiting the AKT/mTOR pathway. Thus, our study shows that CCT020312 may be a potential drug candidate for TNBC treatment.

## Introduction

Breast cancer is one of the most common cancers in women, and triple-negative breast cancer (TNBC) is the most aggressive breast cancer, resulting in a poor outcome because of high molecular heterogeneity and metastatic potential, and a lack of therapeutic targets (Chang-Qing et al., 2019). TNBCs do not express human epidermal growth factor receptor 2 (HER2), estrogen receptor alpha (ERα), or progesterone receptor (PR) (Venema et al., 2019). Due to the absence of these receptors, TNBC does not respond to endocrine therapies, such as tamoxifen, and to HER2-targeting therapies such as herceptin (Jiang et al., 2019). Chemotherapy is still one of the main approaches to treat TNBC. Traditional first-line drugs for treating breast cancer, such as paclitaxel, are not very effective against TNBC. Second-line drugs that are used to treat breast cancer, such as cisplatin, appear to be more effective against TNBC. However, approximately one-third of patients relapse within 3 years of an adjuvant therapy. Therefore, patients with TNBC have a poor prognosis compared with patients with other types of breast cancer (Zhao et al., 2019).

Protein kinase RNA-like endoplasmic reticulum kinase (PERK) plays an important role in the unfolded protein response (UPR) elicited by endoplasmic reticulum (ER) stress (Han et al., 2013; Liu J. et al., 2019). ER stress, due to misfolded protein accumulation in the ER, activates PERK, which subsequently phosphorylates eukaryotic translation initiation factor 2 alpha (eIF2α) and transiently inhibits global protein translation, while selectively increasing the expression of activating transcription factor 4 (ATF4). Subsequently, ATF4 triggers the expression of some genes, including CCAAT-enhancer binding protein (C/EBP) homologous protein transcription factor (*CHOP*), and restores ER homeostasis (Cubillos-Ruiz et al., 2017; Wang et al., 2017; Dadey et al., 2018). The activated PERK pathway directly phosphorylates both nuclear factor (erythroid-derived 2)-like-2 (Nrf2) and forkhead box protein O1 (FOXO) proteins, triggering antioxidant and cell survival mechanisms (He et al., 2001). Additionally, PERK activates AKT signaling to promote cell survival under ER stress conditions (Siwecka et al., 2019). However, under prolonged ER stress, PERK promotes ER stress-induced cell death *via* ATF4/CHOP signaling (Kilberg et al., 2009; Siwecka et al., 2019).

CCT020312 is a selective eIF2α/PERK activator with potent antiproliferative activity at low millimolar concentrations against human colon cancer cells and chemo-sensitizing activity in U-2 OS human osteosarcoma cells (Stockwell et al., 2012). CCT020312 was found to ameliorate progressive supranuclear palsy by increasing the level of phosphorylated PERK and Nrf2 (Bruch et al., 2017). However, the pharmacological effects of CCT020312 have not been comprehensively studied. Hence, we aimed to explore the effects of CCT020312 on TNBC and elucidate its mechanism of action.

## Materials and Methods

### Reagents

CCT020312 was purchased from MedChemExpress (Monmouth Junction, NJ, USA). Female nude mice (aged 5 weeks, weighing 18–22 g) were procured from Beijing HFK Bioscience Co., Ltd. (Beijing, China). Matrigel was purchased from BD Biosciences (Franklin Lakes, NJ, USA). The detailed information of primary antibodies against cyclin-dependent kinase 4 (CDK4), CDK6, cyclin D1, B-cell lymphoma 2 (Bcl-2), Bcl-2-associated X protein (Bax), cleaved poly (ADP-ribose) polymerase (PARP), PERK, phosphorylated PERK (p-PERK), eIF2α, p-eIF2α, ATF4, CHOP, protein kinase B (AKT), p-AKT, mammalian target of rapamycin (mTOR), p-mTOR, and Ki-67 is presented in **Supplementary Table S1**. Goat anti-rabbit immunoglobulin G (IgG) and goat anti-mouse IgG secondary antibody were purchased from Cell Signaling Technology (Danvers, MA, USA). SuperSignal West Femto Trial Kit was purchased from Thermo Fisher Scientific (MA, USA). Cell Counting Kit-8 (CCK-8) was purchased from Bimake (Houston, TX, USA). Enhanced BCA Protein Assay Kit, Cell lysis buffer for western blotting and IP, and Annexin V/fluorescein isothiocyanate (FITC) apoptosis detection kit were purchased from Beyotime (Shanghai, China). *CHOP* RNA interference (RNAi) plasmid was purchased from GenePharma Co., Ltd. (Shanghai, China).

### Cell Culture

Human TNBC cell lines, MDA-MB-453 and CAL-148, were purchased from the American Type Culture Collection (ATCC). The two cell lines were cultured in Leibovitz’s L-15 and RPMI 1640 medium supplemented with 10% fetal bovine serum, respectively. The cells were grown at 37°C in a humidified 5% CO_2_ atmosphere.

### CCK-8 Assay

MDA-MB-453 (8 × 10^3^ cells/well) and CAL-148 cells (4 × 10^3^ cells/well) were seeded in 96-well plates and treated with CCT020312 at different doses for 24 or 48 h. Then, 10 µl of CCK-8 solution was added to each well and incubated for 1 or 2 h at 37°C. The absorbance of the sample was measured at 450 nm using a full wavelength microplate reader (Thermo scientific, MA, USA). The viability of cells was calculated relative to the viability of untreated cells.

### Colony Formation Assay

CAL-148 cells were seeded in six-well plates (500 cells/well) and treated with 0, 4, 6, and 8 μM CCT020312 for 12 days. The colonies were washed three times with cold phosphate-buffered saline (PBS) and fixed in 4% paraformaldehyde for 30 min at room temperature. The surviving colonies were stained with crystal violet for 15 min. The colonies with more than 50 cells were counted under an inverted microscope (Nikon, Tokyo, Japan).

### Real-Time Cell Analysis Using xCELLigence

Cell growth was detected in real time using a well-described system (xCELLigence, Roche, Basel, Switzerland). All xCELLigence plates were seeded with MDA-MB-453 (2 × 10^4^ cells/well) and CAL-148 cells (2 × 10^3^ cells/well) and treated with CCT020312 at different doses on the following day. Cell growth is reported as cell index (CI), which reflects a consistent, logarithmic relationship with the cell number. All experiments were performed in triplicate, and data are presented as mean CI ± standard deviation (SD) over time.

### Western Blotting

Total cell proteins and tissue proteins were extracted with RIPA lysis buffer, and the protein concentration was measured using the Enhanced BCA Protein Assay Kit. Subsequently, the proteins were separated by sodium dodecyl sulfate-polyacrylamide gel electrophoresis and transferred on to polyvinyl difluoride membranes. The polyvinyl difluoride membranes were blocked with 5% BSA and incubated with primary antibodies (**Supplementary Table S1**). Immunoblotting was performed as previously described (Li et al., 2018). Protein bands were visualized using the SuperSignal West Femto Trial Kit and detected using the Tanon 5200 Chemiluminescent Imaging System (Shanghai, China).

### Immunohistochemistry (IHC)

Tumor specimens were fixed in 4% paraformaldehyde, and then embedded in paraffin and sectioned. The sections were deparaffinized/rehydrated. The sections were then immersed in citrate unmasking solution and heated in a microwave for antigen unmasking. After blocking with 50 µl of goat serum for 30 min at room temperature, the sections were incubated overnight with primary antibodies (**Supplementary Table S1**) at 4°C, followed by incubation with secondary antibodies. The signal from each section was visualized with 3’-diaminobenzidine reagent and counterstained with hematoxylin. Images were captured using a light microscope.

### Apoptosis Assay

MDA-MB-453 and CAL-148 cells were seeded in six-well plates (1 × 10^6^ cells/well). On the following day, the cells were treated with CCT020312 at different concentrations and incubated for 24 h. The cells were then trypsinized, washed with PBS, and stained using the Annexin V/FITC kit, according to the manufacturer’s protocol. Apoptosis was detected by flow cytometry using the FACScan flow cytometer (Beckman Coulter, CA, USA). The cell population was separated into the following three groups: live cells with a low level of fluorescence (Annexin V-negative, propidium iodide (PI)-negative), early apoptotic cells with green fluorescence (Annexin V-positive, PI-negative), and necrotic and advanced-stage apoptotic cells with both red and green fluorescence (Annexin V-positive, PI-positive).

### Cell Cycle Analysis

MDA-MB-453 and CAL-148 cells were seeded in six-well plates (1 × 10^6^ cells/well) and treated with CCT020312 at different concentrations on the following day. After 24 h of incubation, the cells were trypsinized, washed with PBS, and fixed in ice-cold 70% ethanol overnight at 4°C. The cells were subsequently washed three times with PBS and incubated with PI and RNase at 37°C for 1 h. Flow cytometry was performed to analyze the cell cycle data according to the manufacturer’s instructions.

### CHOP RNAi Plasmid Transfection *In Vitro*

MDA-MB-453 and CAL-148 cells were seeded in six-well plates (4 × 10^5^ cells/well) and transfected with 2 μg of *CHOP* RNAi plasmid and control RNAi plasmid using Lipofectamine 3000 and Opti-MEM I reduced serum medium for 6 h. After overnight recovery, MDA-MB-453 and CAL-148 cells were treated with 8 or 10 μM CCT020312 for 24 h, and the cells were collected for western blotting.

### Gene Expression Profiling Interactive Analysis (GEPIA) Dataset

GEPIA (http://gepia.cancer-pku.cn/) is a newly developed interactive web server for analyzing RNA sequencing data of tumor and normal samples using a standard processing pipeline. It provides customizable functions such as tumor/normal differential expression analysis, profiling according to cancer types or pathological stages, patient survival analysis, similar gene detection, correlation analysis, and dimensionality reduction analysis (Tang et al., 2017). We used the GEPIA dataset to analyze the AKT/mTOR pathway in breast cancer in relation to CHOP or ATF4, both of which are downstream of PERK.

### Orthotopic Implantation Model

The experiments were performed in accordance with the National Guidelines for Animal Care and Use and approved by the Animal Care and Use Committee of Chongqing Medical University. An orthotopic implantation model was established in nude mice by implanting MDA-MB-453 cells (5 × 10^6^ cells/50 µl) mixed with Matrigel (1:1) in the mammary fat pad. When the tumors grew to approximately 4–5 mm^3^, the mice bearing the xenografts were randomly divided into two groups (n = 5/group) and were intraperitoneally injected with corn oil or 24 mg/kg CCT020312. Tumor growth was measured at 2-day intervals after injection. Tumor volume (V) was measured using a slide caliper and calculated using the following formula: V (mm^3^) = 0.5 × ab^2^, where a and b represent the long diameter and perpendicular short diameter (mm) of the tumor, respectively.

### Statistical Analysis

The data were analyzed with Student’s *t-*test using GraphPad Prism 5.0 software (GraphPad, Inc., Chicago, IL, USA). All data are expressed as mean ± SD. The criterion for statistical significance was *p* < 0.05.

## Results

### CCT020312 Suppressed TNBC Cell Viability

**Figure 1:**
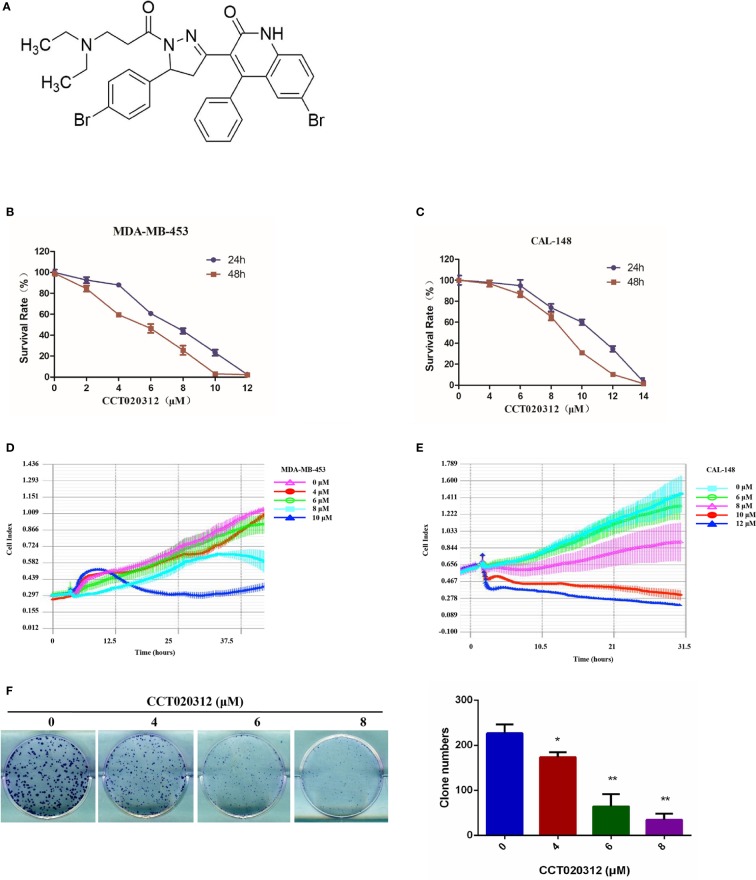
CCT020312 suppressed TNBC cell viability *in vitro*. **(A)** The chemical structure of CCT020312. **(B, C)** MDA-MB-453 cells **(B)** and CAL-148 cells **(C)** were treated with CCT020312 at different doses for 24 or 48 h. Cell viability was measured with CCK-8 (n = 5). **(D, E)** MDA-MB-453 cells **(D)** and CAL-148 cells **(E)** were treated with CCT020312 at different doses, cell growth curve assays were performed using real-time cell analysis by xCELLigence (n = 3). **(F)** CAL-148 cells were treated with various of CCT020312 for 12 days, and the surviving colonies were stained with crystal violet. Colonies with more than 50 cells were counted under an inverted microscope (n = 3). Data are presented as mean ± SD, *p < 0.05 or **p < 0.01 *vs.* control.

The chemical structure of CCT020312 was shown in **Figure 1A**. To examine the effects of CCT020312 on TNBC cells, we treated MDA-MB-453 and CAL-148 cells with the indicated concentrations of CCT020312 and measured cell viability with CCK-8. CCT020312 significantly inhibited cell viability in a dose-dependent manner (**Figures 1B, C**). Moreover, we used a real-time cell analysis system to dynamically monitor the cell growth curves of MDA-MB-453 and CAL-148 cells after CCT020312 treatment. CCT020312 inhibited the proliferation of both cell lines in a dose- and time-dependent manner (**Figures 1D, E**). Furthermore, a colony formation assay showed that 4, 6, and 8 μM CCT020312 significantly inhibited the colony formation of CAL-148 cells in a dose-dependent manner (**Figure 1F**). Thus, the above results demonstrate the *in vitro* anti-TNBC effects of CCT020312.

### CCT020312 Induced the Apoptosis of TNBC Cells

**Figure 2:**
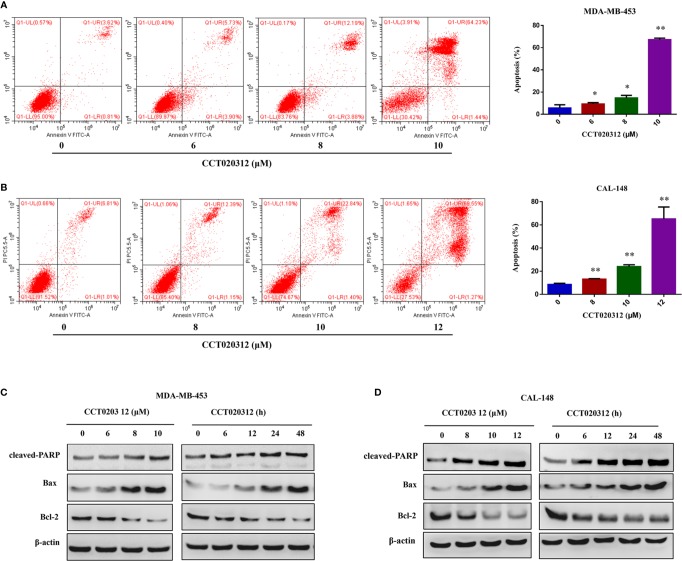
CCT020312 induced the apoptosis of TNBC cells. **(A, B)** MDA-MB-453 cells **(A)** and CAL-148 cells **(B)** were treated with CCT020312 at different concentrations for 24 h. Flow cytometry was used to detect apoptosis (n = 3). Data are presented as mean ± SD, *p < 0.05 or **p < 0.01 *vs.* control. **(C, D)** MDA-MB-453 cells **(C)** and CAL-148 cells **(D)** were treated with various of CCT020312 for 24 h, or treated with 8 and 10 μM CCT020312 for indicated time, then cells were collected to detect cleaved PARP, Bax, and Bcl-2 level using Western blotting.

To examine the effects of CCT020312 on the apoptosis of TNBC cells, flow cytometry was used to detect the percentage of apoptotic cells after the treatment of MDA-MB-453 and CAL-148 cells with 0, 6, 8, 10, and 12 μM CCT020312 for 24 h. The proportion of apoptotic cells in both cell lines increased in a dose-dependent manner (**Figures 2A, B**). Additionally, CCT020312 increased the protein levels of cleaved PARP and the pro-apoptosis protein Bax and decreased the protein levels of the anti-apoptosis protein Bcl-2 in MDA-MB-453 and CAL-148 cells (**Figures 2C, D**).

### CCT020312 Induced G1 Phase Arrest and Modulated Cell-Cycle-Related Proteins in TNBC Cells

**Figure 3:**
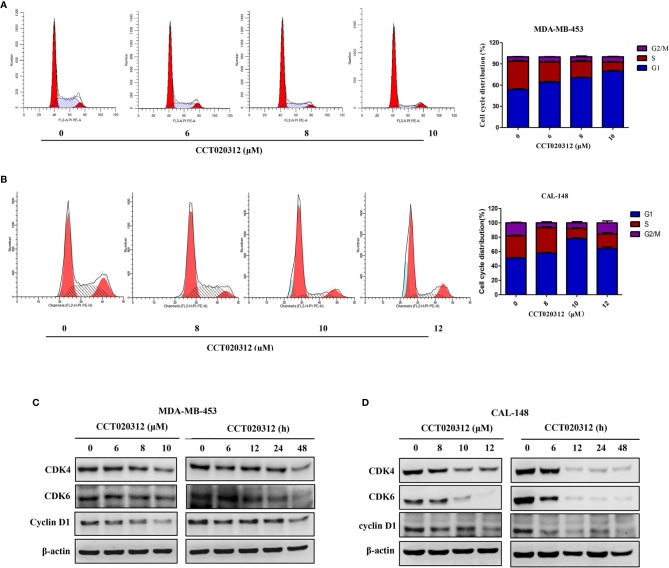
CCT020312 induced G1 phase arrest in TNBC cells. **(A, B)** MDA-MB-453 cells **(A)** and CAL-148 cells **(B)** were treated with CCT020312 at different concentrations for 24 h. The flow cytometry was used to analyze the cell cycle data (n = 3). Data are presented as mean ± SD. **(C, D)** MDA-MB-453 cells **(C)** and CAL-148 cells **(D)** were treated with various of CCT020312 for 24 h, or treated with 8 and 10 μM CCT020312 for indicated time, then cells were collected to detect CDK4, CDK6, and cyclin D1 level using Western blotting.

PI staining followed by flow cytometry was used to detect cell cycle distribution after the treatment of MDA-MB-453 and CAL-148 cells with 6, 8, and 10 μM CCT020312. As shown in **Figure 3A**, the percentages of MDA-MB-453 cells in the G1 phase were 53.70 ± 1.85% (0 μM CCT020312), 64.13 ± 1.86% (6 μM CCT020312), 70.27 ± 1.29%, (8 μM CCT020312), and 79.53 ± 2.28% (10 μM CCT020312), indicating that CCT020312 induced MDA-MB-453 cell-cycle arrest in the G1 phase in a dose-dependent manner. The effects of CCT020312 on CAL-148 cell cycle arrest were similar to those observed in MDA-MB-453 cells (**Figure 3B**).

To explore the molecular action mechanism of CCT020312-induced cell cycle arrest, western blotting was used to detect the levels of CDKs and cyclin D1—proteins known to play important roles in cell cycle progression. The protein levels of CDK4, CDK6, and cyclin D1 in MDA-MB-453 and CAL-148 cells decreased in a dose- and time-dependent manner with CCT020312 treatment (**Figures 3C, D**).

### CCT020312 Activated PERK/eIF2α/ATF4/CHOP Signaling

**Figure 4:**
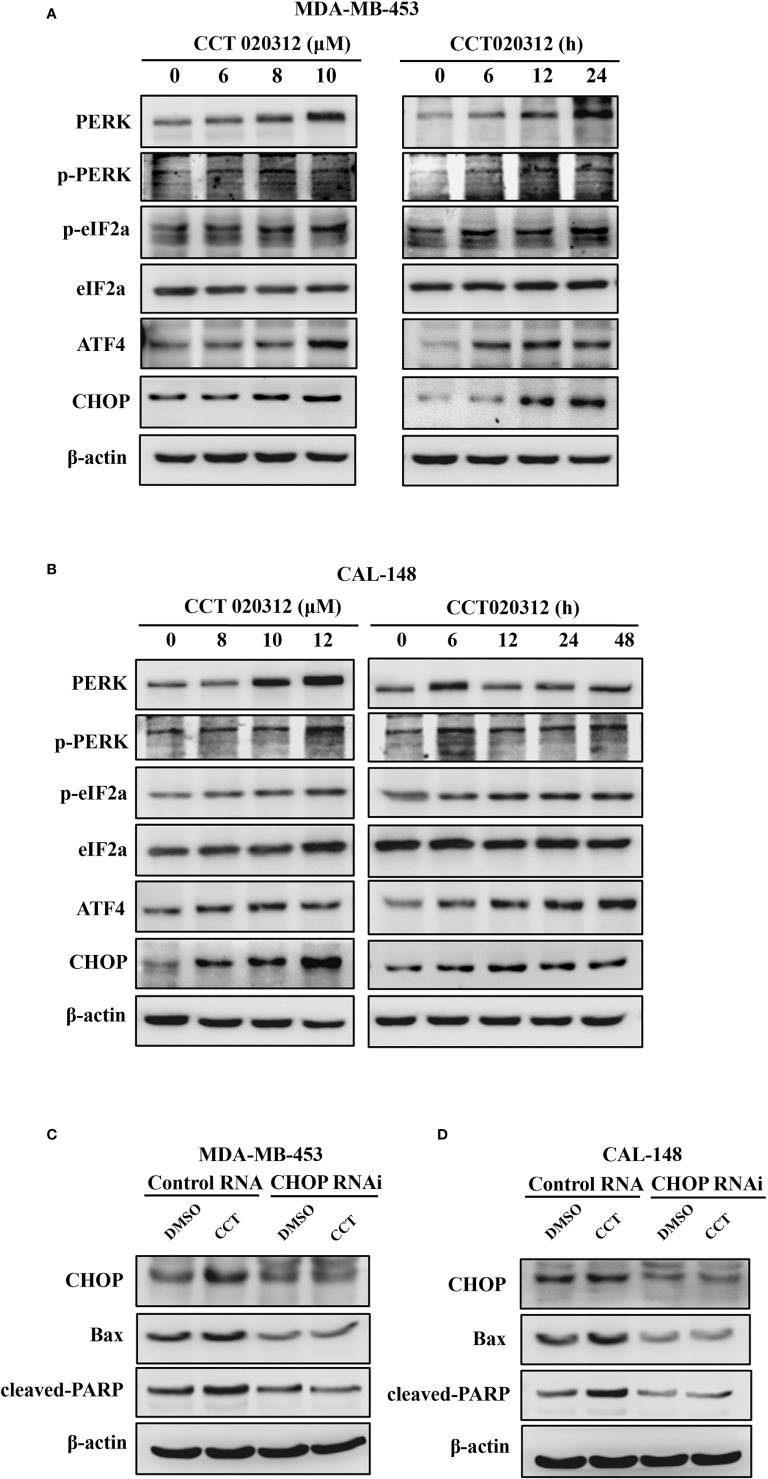
CCT020312 activated PERK/eIF2α/ATF4/CHOP signaling. **(A, B)** MDA-MB-453 cells **(A)** and CAL-148 cells **(B)** were treated with various of CCT020312 for 24 h, or with 8 and 10 μM CCT020312 for indicated time, then cells were collected to detect PERK, p-PERK, eIF2α, p-eIF2α, ATF4, and CHOP level using Western blotting. **(C, D)** MDA-MB-453 cells **(C)** and CAL-148 cells **(D)** were transfected with 2 μg of *CHOP* RNAi plasmid using Lipofectamine 3000 for 6 h. After overnight recovery, MDA-MB-453 and CAL-148 cells were treated with 8 and 10 μM CCT020312 for 24 h, then cells were collected to detect CHOP, cleaved PARP, and Bax level using Western blotting CCT020312 abbreviated as CCT.

CCT020312 is a selective activator of eIF2α/PERK signaling (Bruch et al., 2017). Therefore, we studied the effects of CCT020312 on expression of proteins in the PERK signaling pathway such as eIF2α, ATF4, and CHOP. MDA-MB-453 and CAL-148 cells were treated with CCT020312 at different concentrations. CCT020312 increased the phosphorylation of PERK and eIF2α in a dose- and time-dependent manner; it also increased the ATF4 and CHOP protein levels (**Figures 4A, B**). These results indicate that CCT020312 triggers the activation of PERK signaling in TNBC cells.

CHOP is a major mediator of downstream signaling of PERK, and PERK activation promotes cell death via CHOP signaling. Hence, we explored the role of CHOP in CCT020312-induced apoptosis. We knocked down *CHOP* expression in MDA-MB-453 and CAL-148 cells by RNAi plasmid transfection and treated the cells with CCT020312. Treatment of cells with CCT020312 increased the protein levels of CHOP, cleaved PARP, and Bax. However, treatment of *CHOP-*silenced cells with CCT020312 decreased the protein levels of CHOP, cleaved PARP, and Bax compared with those in CCT020312-treated unsilenced cells (**Figures 4C, D**).

### CCT020312 Suppressed AKT/mTOR Signaling in TNBC Cell Lines

**Figure 5:**
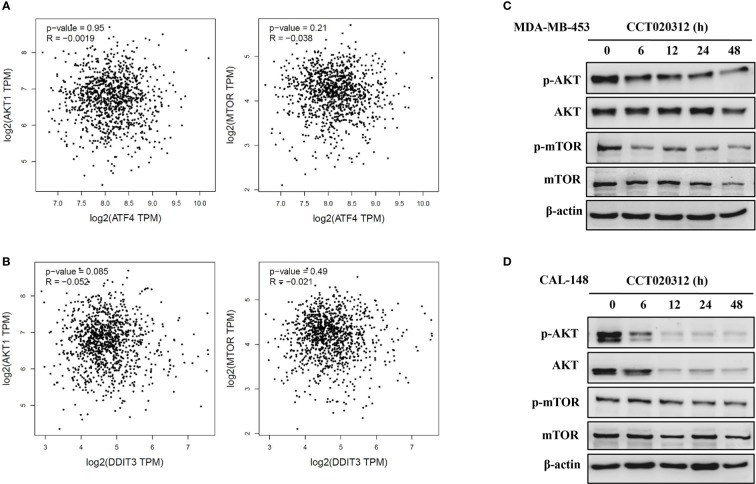
CCT020312 suppressed AKT/mTOR signaling. **(A, B)** GEPIA dataset (http://gepia.cancer-pku.cn/) was used to analyze the AKT/mTOR pathway in breast cancer in relation to ATF4 **(A)** and CHOP **(B)**. CHOP also known as DDIT3. **(C, D)** MDA-MB-453 cells **(C)** and CAL-148 cells **(D)** were treated with 8 and 10 μM CCT020312 for indicated time, then cells were collected to detect AKT, p-AKT, mTOR, and p-mTOR level using Western blotting.

AKT/mTOR pathway inhibition reduces tumor progression. First, we used the GEPIA dataset to study the AKT/mTOR pathway members in breast cancer in relation to CHOP or ATF4, both of which are downstream of PERK. We observed a negative correlation between the AKT/mTOR pathway members and CHOP and ATF4 in breast cancer (**Figures 5A, B**). Next, we examined the effects of CCT020312 on the phosphorylation of AKT and mTOR. The exposure of cells to various concentrations of CCT020312 inhibited the phosphorylation of AKT and mTOR in a time-dependent manner (**Figures 5C, D**). Thus, our results indicate that the inactivation of the AKT/mTOR signaling pathway may be involved in the anti-TNBC effects of CCT020312.

### CCT020312 Inhibited Tumor Growth in an MDA-MB-453 Cell Orthotopic Xenograft Mouse Model

**Figure 6:**
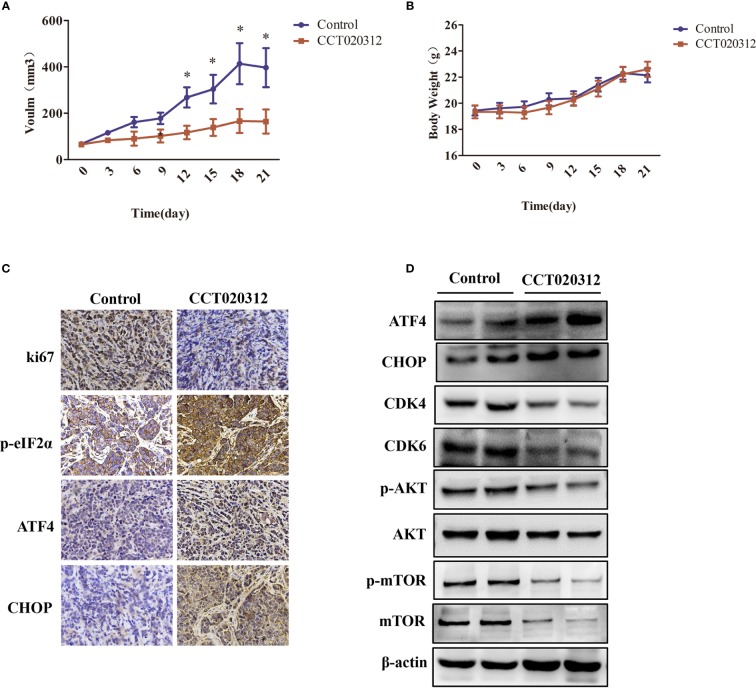
CCT020312 inhibited tumor growth in an MDA-MB-453 cell orthotopic xenograft mouse model. **(A)** Tumor volumes were measured every three days and differed at the end of the treatment period (n = 5). Data are presented as mean ± SD, *p < 0.01 *vs.* control. **(B)** Body weight changes in mice during the 21 days of CCT020312 treatment (n = 5). Data are presented as mean ± SD. **(C)** Representative tumor tissues were sectioned and subjected to immunohistochemistry staining (magnification, × 400). **(D)** Representative tumor tissues from each group were prepared and subjected to Western blotting assay.

An MDA-MB-453 orthotopic xenograft mouse model was established to assess whether CCT020312 inhibits *in vivo* tumor growth. We found that 24 mg/kg CCT020312 inhibited tumor growth after 12 days, and these effects became more apparent after 21 days of CCT020312 treatment (*p* < 0.05) (**Figure 6A**). There was no significant change in the body weight of CCT020312-treated and control mice (**Figure 6B**). IHC revealed that the Ki-67 protein level was significantly reduced in tumor sections from CCT020312-treated mice compared with that from the control mice (**Figure 6C**). These results suggest that CCT020312 has anti-TNBC activity.

To determine whether the activation of the PERK/ eIF2α/ATF4/CHOP signaling pathway and inactivation of the AKT/mTOR signaling pathway were involved in CCT020312-induced tumor inhibition, we performed western blotting and IHC. IHC showed that CCT020312 treatment significantly increased the p-eIF2α, ATF4, and CHOP levels in the xenograft model (**Figure 6C**). This was supported by the Western blotting results (**Figure 6D** and **Supplementary Figure S1**). In addition, Western blotting showed that the treatment with CCT020312 caused a significant decrease in the protein levels of CDK4, CDK6, p-AKT, and p-mTOR (**Figure 6D** and **Supplementary Figure S1**). Thus, CCT020312 inhibited tumor growth via the activation of the PERK/eIF2α/ATF4/CHOP signaling pathway and inactivation of the AKT/mTOR signaling pathway.

## Discussion

To the best of our knowledge, this is the first study to show that CCT020312, a selective activator of eIF2α/PERK, exerts its anti-TNBC effects by inducing G1 phase arrest and apoptosis via the activation of the PERK/eIF2α/ATF4/CHOP pathway and inactivation of the AKT/mTOR pathway. ER stress triggers the UPR in carcinogenesis (Mcgrath et al., 2018; Liu J. et al., 2019; Siwecka et al., 2019). On one hand, ER stress promotes cell survival and induces drug resistance (Rzymski et al., 2009; Pandey et al., 2019; Shi et al., 2019), and on the other hand, long-term ER stress can become pro-apoptotic (Han et al., 2013; Liu Y. S. et al., 2019). UPR consists of three major signaling pathways mediated by inositol-requiring enzyme 1 (IRE-1), ATF6, and PERK. The PERK pathway is a significant determinant of cell fate in UPR (Han et al., 2013; Pakos-Zebrucka et al., 2016; Cubillos-Ruiz et al., 2017; Mohamed et al., 2017). Short-term activation of PERK promotes cell survival *via* the induction of eIF2α phosphorylation (Atkins et al., 2013; Krishnamoorthy et al., 2014). Prolonged activation of PERK triggers death pathways and leads to apoptosis *via* the PERK/eIF2α/ATF4 axis and the subsequent activation of CHOP, also known as growth arrest and DNA damage-inducible protein 153 (GADD153) (Chen et al., 2019a; Siwecka et al., 2019). Thus, targeting PERK, a key molecule in UPR-signaling, may be a novel approach for treating cancers (Mcgrath et al., 2018). As CCT020312 is a selective PERK activator, we first investigated whether CCT020312 activated the PERK/eIF2α/ATF4/CHOP pathway in TNBC. As expected, CCT020312 considerably increased the protein levels of p-eIF2α, ATF4, and CHOP in MDA-MB-453 and CAL-148 cells in a time- and dose-dependent manner.

CCT020312 has been reported to ameliorate progressive supranuclear palsy in mice (Bruch et al., 2017), inhibit the proliferation of human colon cancer cells, and increase chemosensitivity of U-2 OS human osteosarcoma cells (Stockwell et al., 2012). However, the effects of CCT020312 on breast cancer have not been reported. In this study, we demonstrated the *in vitro* anti-TNBC effects of CCT020312. The results of the CCK-8 assay, real-time cell analysis, and colony formation assay showed that CCT020312 reduced MDA-MB-453 and CAL-148 cell viability and proliferation, and colony formation in a dose-dependent manner. Furthermore, CCT020312 induced the apoptosis of MDA-MB-453 and CAL-148 cells as evidenced by the increase in the protein levels of cleaved PARP and Bax and the decrease in the levels of Bcl-2. Additionally, the knockdown of *CHOP*, the main molecule mediating the prolonged activation of PERK-induced apoptosis, attenuated the CCT020312-induced increase in the protein levels of cleaved PARP and Bax. Taken together, these findings suggest that CCT020312 induced apoptosis by triggering PERK signaling and ultimately, activating CHOP.

The uncontrolled G1/S checkpoint plays an important role in the progression of human cancers by allowing inappropriate proliferation and distorting fate-driven cell cycle exit (Abuhammad et al., 2019). The CDK and cyclin D1 complexes are important cell cycle regulators. Cyclin D1 binding to CDK4 and CDK6 drive cell cycle entry and G1 phase progression. The inhibition of cyclin D1 and CDK4/6 has been considered a promising strategy for the treatment of cancers including TNBC (Presti and Quaquarini, 2019; Wang et al., 2019). The activation of PERK/eIF2α signaling has been shown to block the translation of cyclin D1 and CDKs, resulting in cell cycle arrest in the G1 phase. CCT020312 promotes eIF2α phosphorylation to inhibit cell cycle G1/S phase transit (Brewer and Diehl, 2000; Stockwell et al., 2012). Our results show that CCT020312 induced G1 phase arrest and decreased the CDK4, CDK6, and cyclin D1 levels. We also found that persistent activation of PERK/eIF2α signaling by CCT020312 induced cell cycle G1 phase arrest by suppressing the expression of the CDK-cyclin complex.

Targeting the AKT/mTOR pathway has been considered an attractive approach in cancer treatment (Li et al., 2015; Chen et al., 2019b; Wen et al., 2019). PERK and the AKT/mTOR pathway exhibit mutual regulation. Although studies have shown that PERK activates the AKT/mTORC1 signaling pathway to promote cell survival (Bobrovnikova-Marjon et al., 2012; Koga et al., 2015), there are some studies showing that persistent activation of PERK signaling inhibits AKT/mTOR signaling (Du et al., 2003; Ohoka et al., 2005; Appenzeller-Herzog and Hall, 2012). In this study, we observed a negative correlation between members of the AKT/mTOR pathway and CHOP and ATF4 in breast cancer, both of which are downstream of PERK. CCT020312 significantly suppressed the protein levels of p-AKT and p-mTOR in MDA-MB-453 and CAL-148 cells. Taken together, these findings indicate that CCT020312 persistently activated PERK/ATF4/CHOP signaling and inhibited Akt/mTOR signaling.

For the past two decades, the inoculation of tumor tissue from patients or human cancer cell lines into immunodeficient rodents is the main approach to establish subcutaneous or orthotopic transplantation tumors. Xenograft models are a major preclinical screen in the development of novel cancer therapeutics (Cl and Pj, 2007). In the present study, an MDA-MB-453 orthotopic xenograft mouse model was established to evaluate the effects of CCT020312 on tumor growth *in vivo*. CCT020312 suppressed tumor growth and reduced the protein levels of CDK4 and CDK6 in MDA-MB-453 xenograft mice. CCT020312 increased the levels of p-eIF2α, ATF4, and CHOP, and decreased the levels of p-AKT and p-mTOR. Thus, CCT020312, a selective eIF2α/PERK activator, inhibited TNBC viability and proliferation by activating the PERK/eIF2α/ATF4/CHOP pathway and inactivating the AKT/mTOR pathway *in vivo*.

In conclusion, our findings demonstrate that CCT020312 exerts anticancer activity by inducing apoptosis and G1 phase arrest in TNBC cells. The mechanism of this anticancer activity of CCT020312 involves the activation of the PERK/eIF2α/ATF4/CHOP pathway and inactivation of the AKT/mTOR pathway. CCT020312 shows potential to be developed as a therapeutic agent for TNBC. Future studies should explore the efficacy and safety of CCT020312 for TNBC treatment.

## Data Availability Statement

The raw data supporting the conclusions of this article will be made available by the authors, without undue reservation, to any qualified researcher.

## Ethics Statement

All animal experiments were approved by the Chongqing Medical University Animal Subjects Ethics Sub-committee and conducted in accordance with the Institutional Guidelines and Animal Ordinance of the Department of Health.

## Author Contributions

WZ conceived ideas, designed the experiments, performed the data analyses and wrote the manuscript. XL designed the experiments, performed the experiments and data analyses, and wrote the manuscript. XY, DZ, HZ, and LL performed the experiments. WL, XZ, and BC performed the part of the experiments and contributed reagents and materials. All authors read and approved the submitted version.

## Funding

This project is supported by grants from National Natural Science Foundation of China (No. 81874100 and No.81472483), the Science and Technology Project of Chongqing Municipal Education Commission (No.KJQN201800431), Basic Research and Frontier Exploration Project of Yuzhong District of Chongqing (20190102), and Subject Talent Training Program of College of Pharmacy of Chongqing Medical University (No.YXY2019XSGG).

## Conflict of Interest

The authors declare that the research was conducted in the absence of any commercial or financial relationships that could be construed as a potential conflict of interest.
