# Supplementary material for: CCT020312 Inhibits Triple-Negative Breast Cancer Through PERK Pathway-Mediated G1 Phase Cell Cycle Arrest and Apoptosis
Source: Front Pharmacol. 2020 May 19;11:737. doi: 10.3389/fphar.2020.00737 (PMC7250150; doi:10.3389/fphar.2020.00737)
Supplement: Supplementary file 1 [file Image_1.pdf]

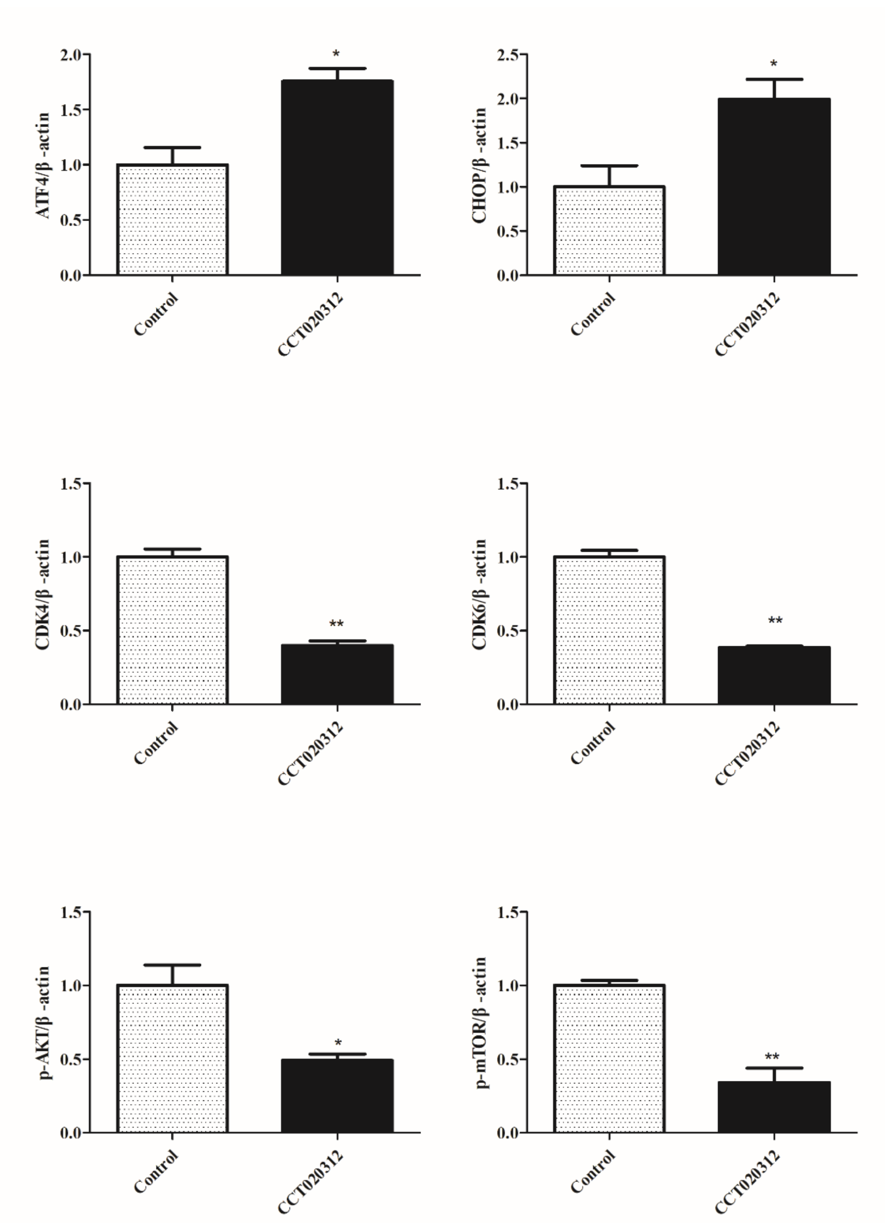

Figure S1. CCT020312 inhibited tumor growth in an MDA-MB-453 cell orthotopic xenograft mouse model. Representative tumor tissues from each group were prepared and subjected to Western Blotting assay. The relative protein expression was qualified by Tanon GIS software and data are presented as mean ± SD (n=3), *$p<$ 0.05 or ** $p<$0.01 *vs.* control.
