# Supplementary material for: CCT020312 Inhibits Triple-Negative Breast Cancer Through PERK Pathway-Mediated G1 Phase Cell Cycle Arrest and Apoptosis
Source: Front Pharmacol. 2020 May 19;11:737. doi: 10.3389/fphar.2020.00737 (PMC7250150; doi:10.3389/fphar.2020.00737)
Supplement: Supplementary file 2 [file Table_1.doc]

**Table S1. Antibodies for Western blotting and Immunohistochemistry**

| **Antibody** | **Company** | **Catalog** | **Dilution ratio** |
| --- | --- | --- | --- |
| CDK4 | Cell Signaling Technology | 12790 | 1:1000 (WB) |
| CDK6 | Cell Signaling Technology | 13331 | 1:1000 (WB) |
| Cyclin D1 | Cell Signaling Technology | 55506 | 1:1000 (WB) |
| Bcl-2 | Affinity Biosciences | AF6139 | 1:1000 (WB) |
| Bax | Cell Signaling Technology | 2772 | 1:1000 (WB) |
| Cleaved PARP | Cell Signaling Technology | 5625 | 1:1000 (WB) |
| PERK | Cell Signaling Technology | 5683 | 1:1000 (WB) |
| Phospho-PERK (Thr982) | Affinity Biosciences | DF7576 | 1:2000 (WB) |
| eIF2α | Cell Signaling Technology | 5324 | 1:1000 (WB) |
| Phospho-eIF2α (Ser51) | Cell Signaling Technology | 3398 | 1:1000/1:100 (WB/IHC) |
| ATF4 | Cell Signaling Technology | 11815 | 1:1000 (WB) |
| CHOP | Cell Signaling Technology | 2895 | 1:1000 (WB) |
| AKT | Cell Signaling Technology | 4691 | 1:1000 (WB) |
| Phospho-Akt (Ser473) | Cell Signaling Technology | 4060 | 1:2000 (WB) |
| mTOR | Cell Signaling Technology | 2983 | 1:1000 (WB) |
| Phospho-mTOR (Ser2448) | Cell Signaling Technology | 5536 | 1:1000 (WB) |
| Ki67 | Affinity Biosciences | AF0198 | 1:200 (IHC) |
| ATF4 | abcam | Ab184909 | 1:100 (IHC) |
| CHOP (DDIT3) | Servicebio | GB11204 | 1:500 (IHC) |
